# Spreading the word: pediatric pain education from treatment to prevention

**DOI:** 10.1186/s40945-022-00151-4

**Published:** 2022-11-15

**Authors:** Kelly Ickmans, Emma Rheel, Juliana Rezende, Felipe José Jandre Reis

**Affiliations:** 1 Faculty of Physical Education and Physiotherapy, Department of Physiotherapy, Human Physiology and Anatomy (KIMA), Vrije Universiteit Brussel, Laarbeeklaan 103 – building B, BE-1090 Brussels, Belgium; 2 Movement & Nutrition for Health & Performance research group (MOVE), Department of Movement and Sport Sciences, Faculty of Physical Education and Physiotherapy, Vrije Universiteit Brussel, Pleinlaan 2, 1050 Brussels, Belgium; 3 Department of Physical Medicine and Physiotherapy, Universitair Ziekenhuis Brussel, Laarbeeklaan 101, 1090 Brussels, Belgium; 4 Department of Experimental-Clinical and Health Psychology, Ghent University, Henri Dunantlaan 2, 9000 Ghent, Belgium; 5 Physical Therapy Department, Instituto Federal do Rio de Janeiro (IFRJ), Rua Professor Carlos Wenceslau, 343, Realengo, Rio de Janeiro, Brazil

**Keywords:** Pain, Education, Communication, Pediatrics, Children, Adolescents, Pain science education

## Abstract

Pain affects everyone hence one can argue that it is in each individual’s interest to understand pain in order to hold correct and adaptive beliefs and attitudes about pain. In addition, chronic pain is reaching pandemic proportions and it is now well known that people living with chronic pain have a reduced life expectancy. To address and to prevent the growth of this public health disaster, we must start looking beyond adulthood. How children view pain has an impact on their behavioral coping responses which in turn predict persistent pain early in the lifespan. In addition, children who suffer from chronic pain and who are not (properly) treated for it before adolescence have an increased risk of having chronic pain during their adult life. Explaining pain to children and youth may have a tremendous impact not only on the individual child suffering from chronic pain but also on society, since the key to stop the pain pandemic may well lie in the first two decades of life. In order to facilitate the acquisition of adaptive behavioral coping responses, pain education aims to shift people’s view on pain from being an apparent threat towards being a compelling perceptual experience generated by the brain that will only arise whenever the conceivable proof of danger to the body is greater than the conceivable proof of safety to the body. Nowadays a lot of pain education material is available for adults, but it is not adapted to children’s developmental stage and therefore little or not suitable for them. An overview of the state-of-the-art pain education material for children and youth is provided here, along with its current and future areas of application as well as challenges to its development and delivery. Research on pediatric pain education is still in its infancy and many questions remain to be answered within this emerging field of investigation.

## Background

Pain is the most common reason for people to consult a healthcare professional, and in the majority of cases that is a physiotherapist [1]. In fact, 90% of patients treated by a physiotherapist report pain [2]. Traditionally, physiotherapists have been trained with a strong biomedical focus, explaining one’s pain by a narrow range of potential contributors such as tissue state and posture [3]. Contemporary pain science has provided physiotherapists with the knowledge that a wider range of potential contributors add to a person’s experience of pain. Essential to that is that pain is a biopsychosocial phenomenon that is undeniably influenced by biological (e.g., nociception, genetics), psychological (e.g., cognitions, beliefs), and social/contextual (e.g., family, friends, culture, school, work) factors. Pain science has taught us that pain is not a signal that originates from bodily tissues, nor a marker of tissue damage or pathology. In contrast, pain is a compelling perceptual experience generated by the brain based upon its evaluation of danger to bodily tissues and its need to protect these tissues [4].

Shifting people’s view on pain from being an apparent threat towards being a brain output that will only arise whenever the conceivable proof of danger to the body is greater than the conceivable proof of safety to the body, is essentially the core objective of pain education. Pain education, also referred to as “pain (neuro)science education” [5, 6], “therapeutic neuroscience education” [7, 8], or “explaining pain” [4], gently guides people through this process of changing their conceptual understanding of pain [9].

Without realizing it, almost everyone experiences pain on a daily basis (e.g., a paper cut, a blister from wearing new shoes, bumping your toe against the coffee table, sore fingers or toes due to freezing outside temperatures, etc.), however many people with persistent pain experience insupportable pain on a daily basis. Indeed, pain is a normal human experience that can become abnormal when it becomes overprotective [10]. One can therefore argue that it is in each individual’s interest to understand pain in order to hold correct and adaptive beliefs and attitudes about pain. Moreover, the experience of pain will trigger certain behavioral coping responses that can predict persistent pain early in the lifespan [11, 12]. Consequently, children who suffer from persistent pain and who’s pain problem is not (properly) managed before adolescence, have an increased risk of having chronic pain during their adult life [13, 14]. Indeed, half to two-thirds of children with chronic pain will grow up to be adults with chronic pain [13, 15]. Thus, knowing that chronic pain is reaching pandemic proportions [16–19] and that people living with chronic pain have a reduced life expectancy [20, 21], there is no denying that chronic pain has become a major public health problem. To address and to prevent the growth of this pandemic we must start looking beyond adulthood. The key to this may well lie in the first two decades of life and the proverb ‘What is learned in the cradle is carried to the grave’ may be a good approach to influence long-term health behavior on a larger scale by teaching children and youth more about pain.

This masterclass has several aims. Firstly, we present which areas currently enable the possibility of applying pediatric pain education in practice. Secondly, we provide a state-of-the-art overview of pain education material for children and adolescents. The third aim of this masterclass is to present some of the many challenges to pediatric pain education. Lastly, it intends to set out lines for future research.

## Main text

Throughout this manuscript, the overarching term “pain education” will be used in order to encompass both the pain *science* education component (i.e., underlying mechanisms explaining one’s pain) as well as the pain *management* education component (i.e., what is one able to do about it?) of pain education.

### Areas of application for pediatric pain education

#### Pain education as a therapeutic intervention

Since its inauguration by Louis Gifford at the International Association on the Study of Pain (IASP) 9th World Congress on Pain in 1999 [22], numerous studies on pain education as a therapeutic tool for adults with chronic pain have been performed, most of which in people with chronic musculoskeletal pain. Overall, the evidence in adults with chronic pain tells us that effect sizes of pain education as a standalone intervention on clinical outcomes are small [23] and its effectiveness is of short duration [24]. In fact, systematic reviews show that combining pain education with a movement-based intervention (e.g., exercise) is superior to education alone [25] or even to exercise alone [26]. Therefore, in order to enhance long term treatment effects for people with chronic pain, it is essential to blend pain education with movement-based interventions, making physiotherapists one of the professionals of choice (i.e., with the most optimal knowledge and skills set) to implement this in clinical practice. Despite this bulk of evidence in adults with chronic pain, to our knowledge only three intervention studies have been conducted on pain education in children with chronic pain [27–29]. Pas et al. [28] piloted a self-developed pediatric pain education program (PNE4Kids) [6] in children aged 6–12 years with functional abdominal pain disorders and demonstrated short-term improvements of ‘usual care + PNE4Kids’ in terms of functional disability, pain-related fear, local pressure pain sensitivity and parental pain catastrophizing. Andias et al. [27] provided proof of concept of a combined approach (i.e., pain education + exercise therapy) in adolescents with chronic idiopathic neck pain by demonstrating its feasibility and beneficial effects compared to a no intervention control group in terms of neck extensors endurance capacity and pain knowledge. In their case series, Selhorst et al. [29] found that a one-time brief pain educational video + a standard physiotherapy program led to immediate reductions in pain catastrophizing and fear-avoidance beliefs and that these reductions were sustained over a period of two weeks in adolescents with subacute or chronic patellofemoral pain.

During the last decade, pain education research in adults has shifted to explore its potential benefits in non-chronic pain states such as acute and subacute low back pain [30, 31] as well as in the run-up to surgical procedures [32–34]. The perioperative application of pain education has the potential to target maladaptive pain cognitions and beliefs such as anxiety and fear of movement after surgery and to improve patient surgical experiences and decrease healthcare utilization [32–34]. Also in the realm of pediatric pain science, Crandall et al. [35] demonstrated some promising beneficial effects of pre-operative pain education, such as that the majority of children receiving pre-operative tonsillectomy pain education reported that it helped them with their postoperative pain.

In summary, pain education as a therapeutic intervention in chronic as well as non-chronic (perioperative) pain states appears to have promising potential to help children in pain.

#### Pain education as a preventive strategy

On the true preventative side, very recently, pain education is being delivered to and studied in a one-on-one format in healthy children [36, 37] as well as in larger groups at middle schools [31, 38–43]. Rheel et al. [36] found that healthy children (aged 8–15 years) who watched a 15- minute pain education video before an experimental heat pain task had higher pain knowledge and higher heat pain thresholds after watching the video compared to a group of children who did not watch this video. However, no group differences were found on experienced pain intensity, pain-related fear and pain catastrophizing during the heat pain task. Bacardit-Pintó et al. [37] mainly explored whether delivering an interactive 45-minute pain education session to healthy children (aged 8–12 years) attended by their parents, would result in improved pain-related outcomes in the parents. After this session parents’ pain knowledge yet also children’s fear of pain reported by their parents improved. No pre-post differences however, were found regarding parental pain catastrophizing or parental pain vigilance and awareness. Studies examining whether delivering pain education to larger groups of children (age range: 10–15 years) in a school/classroom setting (i.e., group sizes ranging from 15 to 70 students) via a lecture and/or a video demonstrate beneficial changes in pain knowledge, healthier beliefs regarding pain [32, 39–44], and ultimately positive behavioral outcomes such as less pain medication use [41]. Positive behavioral results were larger when the initial pain education session was supplemented by short video booster sessions after a few months [41].

Delivering pain education to (healthy) children in the context of upcoming painful procedures (e.g., vaccination) is currently an unexplored research avenue with potential for short- and long-term pain-related (behavioral) outcomes in children. Likewise, given its promising potential upon health-promoting behavior, implementing a dedicated pain education program in the school curriculum needs to be further explored, especially to study true long-term effects (i.e., years later) into adulthood.

### Current evidence of pain education material applied to children and youth

Table 1 provides an overview of the current evidence of pediatric pain education material. It includes covered topics, targeted learning objectives [44], tools for transferring pain concepts (e.g., metaphors, storytelling, illustrations, …), and delivery methods (e.g., face-to-face, via a video, via a book(let), using a PowerPoint presentation, using a board game, …) for all available material. Furthermore, if the material was tested in a trial, the targeted study participants and the professional background of the educator were indicated. The results of the different trials were discussed under the previous subheading (i.e., ‘Areas of application for pediatric pain education’).

**Table 1:** Overview of pain education material applied to children and youth

**Interactive**^**a**^ **pain education material**							
**Author, year**	**Covered topics**	**Learning objectives (according to Leake et al.** [44]**)**^b^	**Education material**	**Tool(s) (e.g., metaphors, illustrations) for transferring concepts**	**Delivery method(s)**	**Educator(s)**	**Target(ed) audience**
Pas et al. (2018) [6] (2020) [28]	■ PNE4Kids is divided in 3 sections: ‣■ The pain system and its function (i.e., nervous system anatomy, from nociception to pain, modulation of nociceptive information) ‣■ Bioplasticity and persistent pain ‣■ Therapeutic strategies to ease pain with application to the child’s own situation / daily life	(1)–(6)	PNE4Kids toolbox consisting of: ■ A game board ■ 5 privates ■ 1 lieutenant ■ 1 general ■ 5 cubes/messages ■ 5 computers ■ 1 brain ■ 1 spinal cord ■ PNE4Kids instructions for educator Currently available in English, Dutch, and Danish via http://www.paininmotion.be/pne4kids	■ Allegory of the military ■ Metaphors for brain, spinal cord, peripheral nerves and (danger) messages ■ Examples ■ Storytelling	■ Interactive session(s) with the PNE4Kids toolbox that stimulates children to come up with personal examples and to demonstrate and explain pain themselves. ■ 2 session of ±30 min [6] ■ 1 session of ±60 min [28]	Physiotherapist	■ Healthy children between 6 and 12 years old (and their parents/caregivers) [6] ■ Children between 6 and 12 years old with a diagnosis of functional abdominal pain disorder (and their parents/caregivers) [28]
**Non-interactive**^a^ **pain education material**							
**Author, year**	**Covered topics**	**Learning objectives (according to Leake et al.** [44]**)**^b^	**Education material**	**Tool(s) (e.g., metaphors, illustrations) for transferring concepts**	**Delivery method(s)**	**Educator(s)**	**Target(ed) audience**
Selhorst et al. (2020) [29]	■ Pain-related fear and pain catastrophizing using the “Common Sense Model of Self-Regulation” framework ■ Information on how the body processes nociception, experiences pain ■ The concept that pain does not always mean tissues are being damaged	Not specifically reported but derived by the authors of this masterclass from the reported topics covered: (1), (3)–(5)	■ Psychologically-informed video including pain neuroscience education created by a physical therapist and clinical psychologist using a narrator	Not specifically reported. The article states that adults pain neuroscience education was modified for the adolescent population according to Robins et al. (2016) [45] and tailored to patellofemoral pain	■ Video with a duration of 8 minutes and 30 seconds ■ The video was shown once	Not mentioned	Adolescents with patellofemoral pain between 12 and 17 years old (mean (±SD) age: 14.1 (2.4) years)
Rheel et al. (2021) [36]	■ The pain system and its function: ■ Nervous system anatomy ■ From nociception to pain ■ Modulation of nociceptive information	(1), (3)–(5)	■ Engaging video based on PNE4Kids [6] ■ Children are invited to use the materials from the PNE4Kids toolbox while watching the video	■ Allegory of the military ■ Metaphors for brain, spinal cord, peripheral nerves and (danger) messages ■ Examples ■ Storytelling	■ 15-minute engaging video ■ The video was shown once	Physiotherapist	Children/adolescents between 8 and 15 years old (mean (±SD) age: 12.02 (1.87) years)
Reis et al. (2021) [46]	■ Characterization of pain as the alarm system of the body ■ Pain neurophysiology (neurons, nociceptive system, nociceptive pathways, up- and down-regulation of the nervous system, peripheral and central sensitization) ■ Cognitive, emotional, and behavioral factors that might contribute to pain ■ A summary of the most important concepts in the book ■ True or false quiz consisting of affirmative statements about pain	(1)–(5)	A comic book “A journey to learn about pain”. Currently available in pdf, flipbook, and e-book in English, Portuguese and German via http://pesquisaemdor.com.br/?page_id=84	■ Sequence of illustrations with accompanying speech bubbles ■ Story with different characters ■ Metaphors ■ Examples	■ 40 page comic book ■ Children may read the book themselves or let someone else read it for them (e.g., parent, healthcare professional, …)	N/A	Children between 8 and 12 years old (and their parents/caregivers)
Louw et al. (2020) [41]	■ Peripheral sensitization ■ Central sensitization ■ Bio-psycho-social factors associated with pain ■ Threat appraisal of the brain ■ Nociception, stress, and endocrine responses in pain ■ Various therapeutic endogenous strategies to ease pain	Not specifically reported but derived by the authors of this masterclass from the reported topics covered: (1)–(7)	■ PowerPoint presentation with encouragement to ask questions afterwards ■ 2 follow-up video-presentations	■ Illustrations ■ Metaphors ■ Examples	■ 30-minute, 32-slide PowerPoint presentation delivered in a classroom setting with room for asking questions after the presentation ■ 2 booster sessions at 2 and 4 months follow-up via video-presentation (± 10 min each)	Physiotherapist or occupational therapist	Children of grade 7 in the USA education system (mean age: 12.2 years)
Louw et al. (2019a) [47]	■ Sensitization of the nervous system during a pain experience leading to decreased pain thresholds for movement, emotions and activity ■ Strategies to dampen down a sensitive nervous system	Not specifically reported but derived by the authors of this masterclass from the reported topics covered: (1)–(7)	PNE was delivered via a virtual reality system, including a headset and earphones	■ Illustrations ■ Metaphors ■ Sound	■ 3 sessions delivered 1 week apart ■ Time per session: session 1 (15 min), session 2 (22 min), session 3 (26 min)	Not mentioned	18-year-old high school girl who developed chronic neck pain following a motor vehicle collision 10 months ago
Louw et al. (2018) [38] (2019b) [39] (2020) [41] Podolak et al. (2019) [40]	■ Peripheral sensitization ■ Central sensitization ■ Bio-psycho-social factors associated with pain ■ Threat appraisal of the brain ■ Nociception, stress, and endocrine responses in pain ■ Various therapeutic endogenous strategies to ease pain	Not specifically reported but derived by the authors of this masterclass from the reported topics covered: (1)–(7)	PowerPoint presentation with encouragement to ask questions afterwards	■ Illustrations ■ Metaphors ■ Examples	■ 30-minute, 32-slide PowerPoint presentation delivered in a classroom setting with rooms for asking questions after the presentation [38–41] ■ Class size < 30 students [38, 40] ■ Mean class size = 34 students [39]	■ Physiotherapists [38, 40] ■ Physiotherapist or occupational therapist [39, 41]	■ Children of grades 5–8 in the USA education system (mean (±SD) age: 12.74 (1.13) years) [38] system (mean (±SD) age: 12.8 (1.1) years) [40] ■ Children of grade 7 in the USA education system (mean age: 12.3 years) [39, 41]
Crandall et al. (2008) [35]	■ An introduction describing how each person experiences pain differently ■ Definition of pain and associated feelings ■ Questions the nurses and doctors would ask about their pain (i.e., is there pain, how much, location and were the medicines helping), ■ A review of a 0—10 pain intensity scale ■ Expected location and duration of pain ■ Available pain medications, their helpfulness and side effects ■ Non-pharmacologic pain management strategies (e.g., resting, use of distraction, and the importance of drinking including the drinking of cold liquids and food) ■ To take the pain medicine every 4 h if pain occurs ■ To tell their parents, nurse or doctor if the pain medicine is not working.	Not specifically reported but derived by the authors of this masterclass from the reported topics covered: (5)	Not specifically reported: at least a booklet containing textual information	Not specifically reported	■ During the pre-operative clinic visit with the parent(s) present, children were given the option to either read the booklet themselves or have the researcher read it ■ The booklet was sent to the child’s home afterwards	No educator or a researcher (professional background of researcher was not reported)	Children between 7 and 13 years scheduled for tonsillectomy with or without adenoidectomy (mean (±SD) age: 10.1 (2.2) years)
Wager et al. (2018) [42] Kisling et al. (2021) [43]	The video is divided into three parts: ■ General knowledge on pain (i.e., biological facts on pain) ■ Chronic pain development and maintenance (i.e., how pain is caused and processed by the brain, as well as how chronic pain originates, including examples of psychosocial factors that can contribute to pain) ■ Strategies for the management of chronic pain	(1)–(7)	A 10 min video available on Youtube https://www.youtube.com/watch?v=eJ8THITj_2Y	■ Simple language and catchy illustrations and hand-painted scenes drawn on a white-board. ■ Story is told by a teenage girl ■ Pictures of a male and female character are equally embedded + a sex-neutral character – a ‘brain’ with arms and legs – is included as well	■ Children watched the video once in groups of 15 to 20 [42]. ■ Children watched the video at least 1 time and received a QR-code which allowed them to watch it again if they wanted to [43].	N/A	Children of grades 5–7 in the German education system (age range 10–15 years) ■ (mean (±SD) age: 11.7 (1.1) years) [42] ■ (mean (±SD) age intervention group: 11.21 (0.93) years) [43]
**Mixed (interactive and non-interactive)**^a^ **pain education material**							
**Author, year**	**Covered topics**	**Learning objectives (according to Leake et al.** [44]**)**^b^	**Education material**	**Tool(s) (e.g., metaphors, illustrations) for transferring concepts**	**Delivery method(s)**	**Educator(s)**	**Target(ed) audience**
Andias et al. (2018) [27]	■ The neurophysiology of pain ■ Transition from acute to chronic pain ■ The nervous system’s ability to modulate the pain experience.	Not specifically reported but derived by the authors of this masterclass from the reported topics covered: (1)–(4)	■ Pictures and diagrams (based on Butler & Moseley [48] and Louw & Puentedura [49]) ■ A booklet for use between sessions	Not specifically reported	■ 4 group sessions ■ Groups of 4 to 7 adolescents ■ Time allocated to pain science education decreased from session 1 (45 min) to session 4 (15 min)	Physiotherapists	Adolescents from a secondary school in Portugal (mean (±SD) age intervention group: 17.4 (1.4) years)

^a^The interaction model of communication describes communication as: “a process in which participants alternate positions as sender and receiver and generate meaning by sending messages and receiving feedback within physical and psychological contexts” [50]. Interactive here means that communication is inherent to the pain education material

^b^Targeted learning objectives (according to Leake et al. [44]): (1) Pain is a protector; (2) The pain system can become overprotective; (3) Pain is a brain output; (4) Pain is not an accurate marker of tissue state; (5) There are many potential contributors to anyone’s pain; (6) We are all bioplastic; (7) Pain education is treatment

For the scope of this masterclass, pediatric pain education material was subdivided in three categories: (1) interactive, (2) non-interactive, and (3) mixed (a combination of interactive and non-interactive) pain education material. ‘Interactive’ here means that communication is an inherent part of the pain education material, and *communication* is described as “*a process in which participants alternate positions as sender and receiver and generate meaning by sending messages and receiving feedback within physical and psychological contexts*” according to the interaction model of communication [50]. Yet, there is no available research indicating whether one modality is superior than another. Indeed, the most optimal modalities of providing pain education will depend on several aspects, including target population, group size, setting, etcetera.

**Fig. 1:**
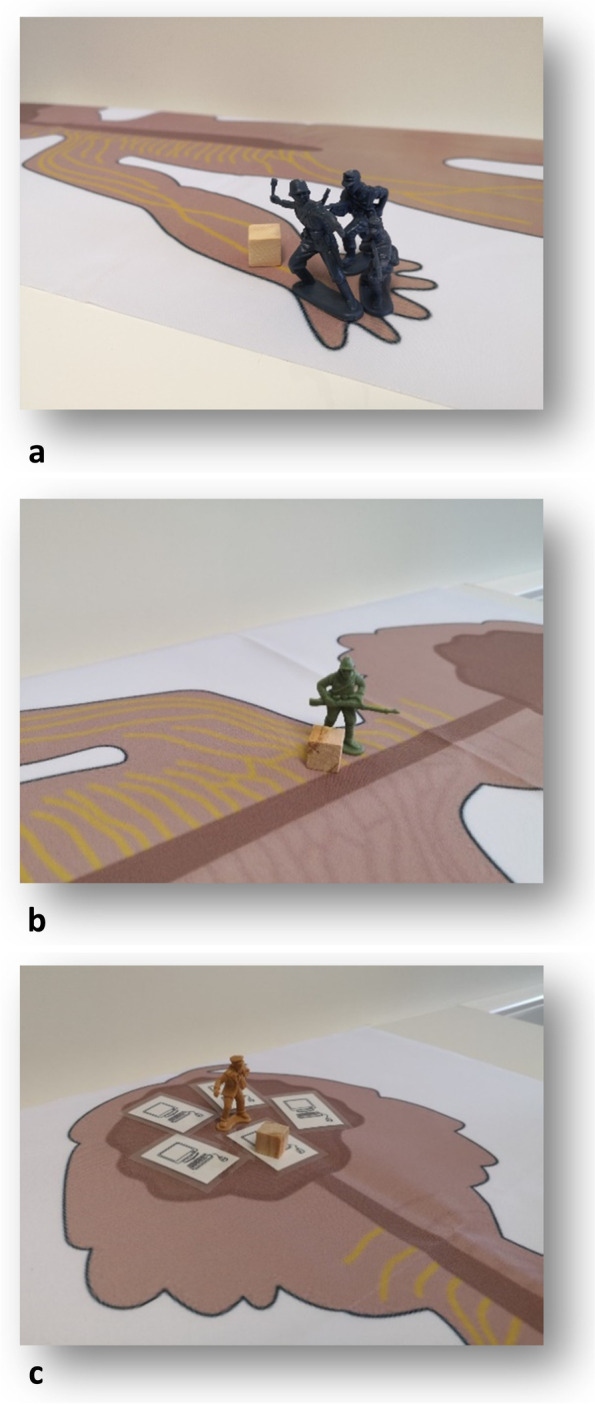
**a** Privates located at the hand and sending a danger message (cube). **b** Lieutenant controlling the elevator and sending messages (cubes) up- and downwards. **c** General in the computer room. Adapted and reprinted with permission from http://www.paininmotion.be/pne4kids [6]

**Fig. 2:**
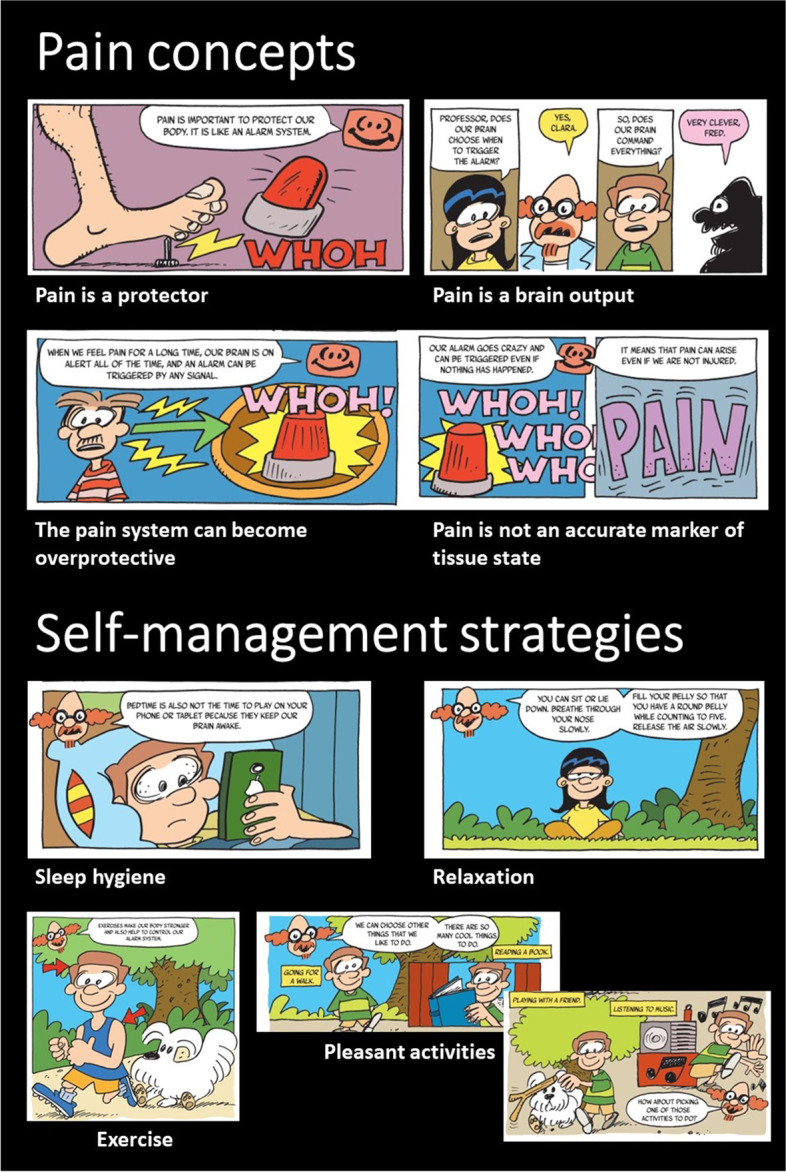
Fragments from the comic book “A journey to learn about pain”. Examples of metaphors used to transfer pain concepts and pain management education. Reprinted with permission from Reis et al. 47

**Fig. 3:**
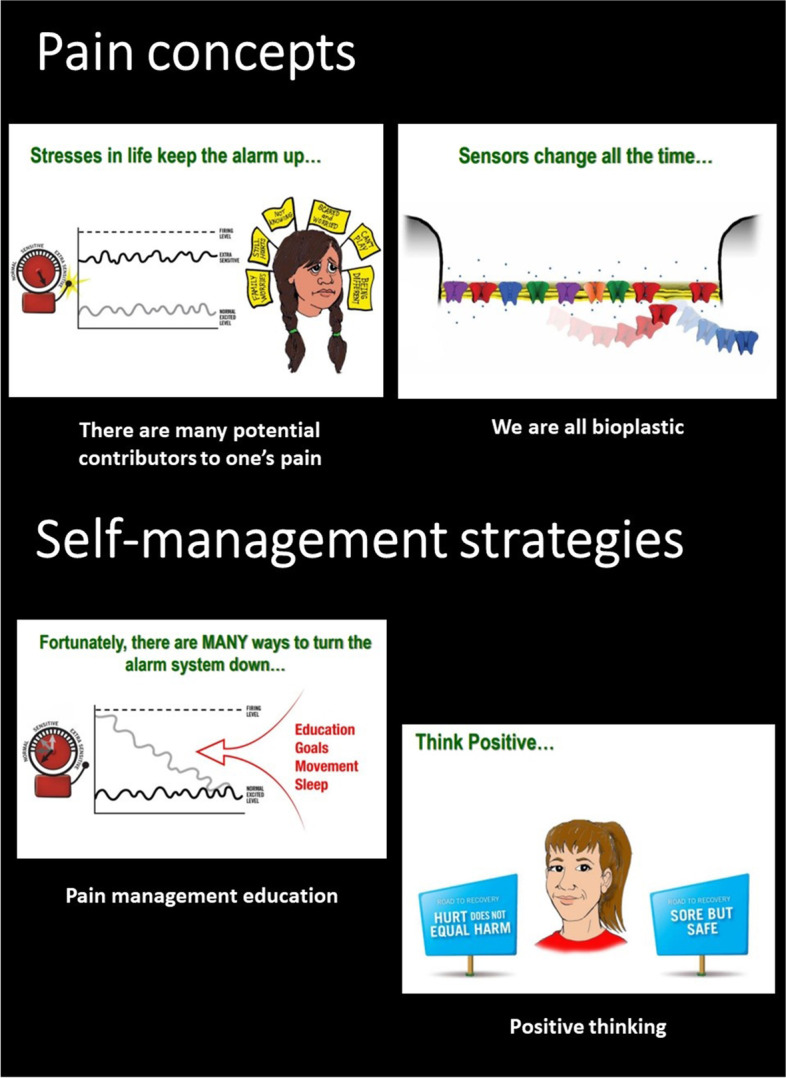
PowerPoint slides from the presentation used to teach middle school children in Louw et al. [38, 39, 41] and Podolak et al. [40]. Examples of illustrations and metaphors used to transfer pain concepts, and pain management education. *Published with permission*

Below we provide some fragments from existing pediatric pain education material that can serve as examples of metaphors, illustrations, and ways of communicating. An example of the metaphors and communication used in PNE4Kids [6] is provided in Table 2, Table 3 and Fig. 1a-d. Figure 2 depicts some fragments from the comic book “A journey to learn about pain” which is developed for children between 8 and 12 years old and their parents/caregivers [46]. The latter two pain education materials are available online for free and in different languages (see Table 1 for URLs). Figure 3 pictures some of the PowerPoint slides that were used in the presentation to explain pain to middle school students in Louw et al. [38, 39, 41] and Podolak et al. [40].

**Table 2:** Metaphors used during PNE4Kids. Adapted and reprinted with permission from http://www.paininmotion.be/pne4kids [6]

Neurophysiological term	Metaphor
Brain	Computer room
Spinal cord	Elevator
Peripheral nerves	Electrical cables
Pain system	The military
Nociceptors	Privates
Neurochemicals within the synaptic cleft	Lieutenant
Thalamus	General
(Non-)noxious signals	(Non-)danger messages

**Table 3:** Communication and use of metaphors to explain nociceptive pain. Adapted and reprinted with permission from http://www.paininmotion.be/pne4kids [6]

*Therapist: “Have you ever cut your finger while helping your parents with cooking? Or have you ever scraped your knee in a fall?”* *Child: “Yes, I once fell with my bike and had a big injury on my knee.”* *Therapist: “And did that hurt?”* *Child: “Yes, very much!”* *Therapist: “Well, I’ll explain to you what happens in your body from the moment your knee was damaged to the moment you actually felt pain.* *When your skin is damaged, the privates at the beginning of the electrical cables wake up and multiply (*Fig. 1a*). As these privates detect potential danger, a message is sent* via *the electrical cables to the lieutenant who is positioned at the elevator (*Fig. 1b*).* *The lieutenant who receives the message looks at it and then decides whether or not the message is important enough to forward to the general in the computer room (*Fig. 1c*). When the lieutenant decides that the message is important enough, he contacts the general* via *his walkie-talkie to ask if the message can be sent* via *the elevator to the computer room. If the general is not too busy, he tells the lieutenant that he can receive one or more messages.* *When the message arrives in the computer room and the general is still not too busy (*e.g. *processing other messages from other parts of the body), he will put the message into several different computers so that the its content can be analyzed in more detail. Each computer has its own task in this analysis; one computer checks from which region the message comes (*e.g.*, your knee or your finger), the other checks whether a similar message was previously received. Another computer analyzes the possible cause (*e.g.*, you drove down a slope too fast). Again another one looks at the environment you are in at the moment (*e.g.*, in the middle of a busy road or in a quiet place in the woods), and another one looks at the possible consequences, such as what thoughts and feelings you experienced along with a similar message (*e.g.*, anger, sadness, shame, …) and what you then did (*e.g.*, cry, scream, laugh away, refuse to get back on your bike or you got back on your bike very quickly, …). When the general, based on the analyses of the computers, is convinced that the message is dangerous, he will create pain. That’s the moment you felt pain.”*

### Challenges in pediatric pain education

#### Developmental considerations

Children’s cognitive, emotional, and physical capabilities change during the entire childhood period. Children perceive the world and how it affects them in substantially different ways compared to adults [51]. Indeed, children emotionally and cognitively appraise pain, its potential implications and how to cope with it unlike adults [52, 53]. For example, children are still learning to master their emotional responses to events, like controlling aggressive, depressive and anxious emotions [52] and have fewer problem solving skills [54]. Therefore, they are often depending on and being helped to cope with threat by parents or other involved adults [52]. Because a child’s cognitive developmental stage may affect their ability to understand and communicate about abstract concepts such as pain, pain education should be tailored to the emotional and cognitive developmental stage of the child concerned. In this regard, age can function as a helpful and convenient guideline and, while only being a rough approximation of a child’s development, it is often the most appropriate proxy [55].

Further, given the complexity of various biopsychosocial developments throughout childhood [56], assessing pain-related outcomes in younger children is not straightforward and evaluating the effectiveness of pain education interventions in children can be challenging. For example, 8 years is the youngest age a child can validly and reliably self-report [57], and only during adolescence, the development of new cognitive skills, like abstract thinking capacities, takes place [58]. Parent proxy reports might be helpful here to examine the effectiveness of pain education interventions in the youngest children.

#### The role of parents

Parents are considered essential participants in the management of their child’s pain. Complex processes and individual factors mediate parents’ and children’s emotional, cognitive, and behavioral responses to pain, which in turn impact the child’s overall functioning [59–61]. Within the Interpersonal Fear Avoidance Model of Pain, parents construe their child’s pain expression from a perspective of own catastrophic appraisals and pain-related fears. By doing so, some tend to engage more in parenting behaviors that may result in adverse consequences for the child (i.e., parental protective behaviors leading to imposed and/or self-chosen activity avoidance). Indeed, certain parental cognitions (e.g., catastrophizing, fears related to their child’s pain) and affective responses towards their child (e.g., criticism, increased attention to pain, granting of special privileges) are associated with maladaptive pain-related outcomes in children [60, 62–64]. Additionally, behavioral responses of parents (e.g., being overly protective, minimizing or magnifying symptoms) relate to poorer child outcomes as well, such as higher pain intensity and functional disability [65–67]. In contrast, other cognitive and behavioral responses, such as parental psychological flexibility and pain acceptance, may provide resilience by promoting child functioning through their impact on both child coping and parenting behaviors [68–70].

In addition, even in the absence of a child’s painful experience or chronic pain state, parents exert a powerful impact on their children’s coping responses to painful events. According to Bandura’s social learning theory, children may learn an entire repertoire of pain responses from their parents through observing and imitating what they have observed [71]. Children witnessing parents who hold strong beliefs about a high threat value of own (persistent) pain, may be more at risk to (chronic) pain vulnerability and related disability. Such maladaptive beliefs may lead to parental modeling of maladaptive pain coping behaviors (e.g., activity avoidance because of the inappropriate belief that activity is dangerous) [59, 72, 73].

Given the bulk of evidence on the influence of parental cognitive, affective, and behavioral responses regarding their child’s pain experience and associated functioning, it is key to assess and address influential pain-related cognitions and beliefs in parents to ensure successful outcomes [62]. Therefore, it seems crucial to at least involve parents in pediatric pain education to optimize child pain-related outcomes. Besides, it seems appropriate to investigate whether certain parents require additional pain education that focuses on their own pain beliefs, perceptions and pain coping behavior as part of their child’s pain management. So far, only Bacardit-Pintó et al. [37] investigated the effects upon several parental outcomes of a pediatric pain education session that was delivered to the child and also attended by the parents. Significant short-term (i.e., 1 week post intervention) improvements were found on parents’ pain knowledge and parent-proxy reports about the child’s fear of pain, highlighting the need for further investigation regarding parental involvement in pediatric pain education to target parental as well as children’s pain-related cognitions and beliefs.

#### Cultural aspects

Within the realm of pain science, social factors have received considerably less coverage in the literature in comparison with psychological and biological factors. The social component of the biopsychosocial model tends to be considered under a narrow perspective by researchers and clinicians, putting aside relevant aspects capable of contributing to pain such as culture, religiosity/spirituality, gender, race, ethnicity, the socio-economic gradient in health, etcetera [74].

The most commonly used proxy measures of culture in pain research are ethnicity/race and country of origin [75]. However, culture is defined by the United Nations Educational, Scientific and Cultural Organization (UNESCO) as “*the set of distinctive spiritual, material, intellectual and emotional features of society or a social group, that encompasses, not only art and literature, but lifestyles, ways of living together, value systems, traditions and beliefs*” [76]. Culture is passed on primarily by the family to the developing child, and it is further influenced later in life by various social institutions (e.g., school, church, and/or other religious or community institutions), all of which have a major impact on the child’s psychological, emotional, and cognitive development [77]. In addition, (young) people can identify themselves belonging to more than one social group, based on ethnicity, gender, nationality, common interests, or sexual orientation [78].

Several pain education tools (e.g., online videos, (comic) books, PowerPoint presentations, games, mobile apps, etc.), including methods for transferring pain concepts such as certain metaphors and illustrations, have been developed in the Western World for adults as well as for children. However, we cannot simply assume that these materials, tools and methods are suitable for all Western contemporaries, let alone for non-Western peers (e.g., a child must also be able to identify himself with the physical traits of the characters used in the education session). Indeed, the meaning, evaluation and interpretation of pain, and the consequent emotional and behavioral coping responses are known to be influenced by culture [79, 80]. A few culturally adapted pain education materials have recently been developed and tested in adults with promising results for poor and low-literate populations and for recent immigrants who are not acculturated yet [81–84]. However, cultural disparities also exist between children and culturally adapted pain education material for children is still non-existent today.

### Emerging lines for future research

Given the bulk of evidence in adult chronic pain management [25, 26] and the proof of concept demonstrated in adolescents with musculoskeletal pain [27, 29], high quality clinical trials are now needed to examine the blending of pain education with movement-based interventions in pediatric chronic pain populations in order to enhance treatment outcomes. In addition, we believe that pediatric chronic pain management requires a multimodal and individually tailored best practice approach and therefore it may be beneficial to explore the possible added value of combining pain education with other individually tailored best practice approaches such as stress management, sleep hygiene, dietary interventions, and other lifestyle approaches.

In addition, there is also need to explore the perioperative application of pain education in children. Likewise, delivering pain education to (healthy) children in the context of skin-breaking procedures seems like a promising avenue of research in order to explore its potential beneficial impact upon short- and long-term pain-related outcomes, especially given its topicality within the worldwide CoViD-19 vaccination campaign. In doing this, effort should be made to properly adapt the content to the forthcoming procedure.

As indicated in the challenges above and given the impact of parental attitudes, responses, and beliefs regarding pain, it seems worthwhile to investigate whether certain parents, besides attending their child’s pain education session(s), also require additional pain education that focuses on their own pain beliefs, perceptions and pain coping behavior as part of their child’s pain management.

Schools also seem to play an important role in addressing and preventing the further growth of the pain pandemic. A recent public health trial demonstrated positive behavioral results, including less use of pain medication 6 months after a 30-minute pain education presentation followed by two video-delivered booster sessions all delivered at school in a classroom setting [41]. In order to influence long-term health behavior, pre-emptive mass pain education by implementing a dedicated pain education program in the school curriculum needs to be further explored, especially to study true long-term effects (i.e., years later) into adulthood.

Lastly, there is also need to develop and test culture-sensitive pediatric pain education materials as well as material that is adapted and tailored to specific patient populations suffering from (chronic) pain such as for instance children living with and beyond cancer or children with neurological disorders. The latter populations will also benefit from understanding their pain, however unique challenges will arise with each of these groups (e.g., the complex relationship between pain and threat in cancer (survivors) and the cognitive ability of children with certain neurological disorders).

**Fig. 4:**
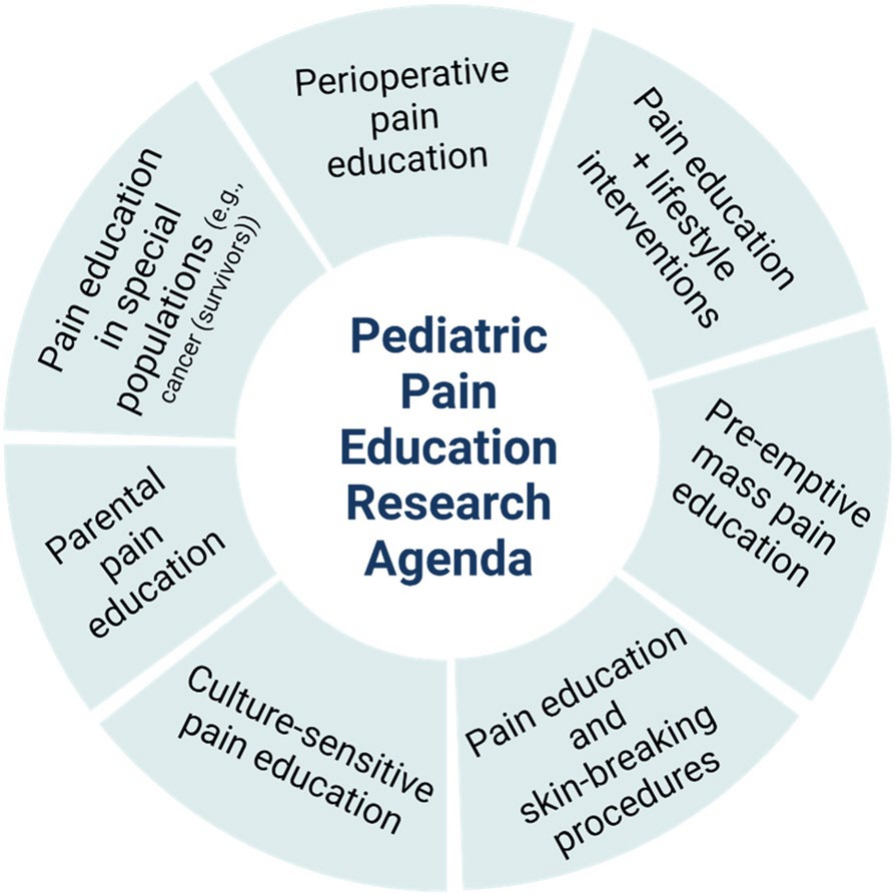
Promising directions for future pediatric pain education research. Created with BioRender.com

Figure 4 pictures a summary of promising directions for future pediatric pain education research.

## Conclusions

Pain education has been widely investigated and implemented in adult chronic pain management and is now receiving growing interest in the realm of pediatric pain. This masterclass provides a thorough overview of available and/or tested pediatric pain education material and its current and future areas of application as well as challenges to its development and delivery. However, research on pediatric pain education is still in its infancy, and it constitutes an emerging field for investigation with tremendous opportunities for the physiotherapy profession. Preventing children from growing up to become adults with chronic pain might be a life-saving procedure; it is our professional duty to spread the word.

## Data Availability

Not applicable.

## Abbreviations

IASP: International Association on the Study of Pain
UNESCO: United Nations Educational, Scientific and Cultural Organization
